# Correction to “Association between the duration of progesterone supplementation treatment and premature neonates outcomes: A retrospective cohort study”

**DOI:** 10.1002/hsr2.1768

**Published:** 2023-12-14

1

Kazemi Aski S, Sharami SH, KabodMehri R, Rahnemaei FA, Milani F, Sabetghadam S. Association between the duration of progesterone supplementation treatment and premature neonates outcomes: a retrospective cohort study. Health Sci Rep. 2023;6:e1721. doi:10.1002/hsr2.1721

The Ethical approval section for the article is corrected as “This study was approved by Guilan University of Medical Sciences with the ethical code IR. GUMS. REC.1401.075. Also, the authors claim that this study complies with the 1975 Declaration of Helsinki, as revised in 2008 (informed consent).”

We apologize for this error.

